# Plant disease classification in the wild using vision transformers and mixture of experts

**DOI:** 10.3389/fpls.2025.1522985

**Published:** 2025-06-18

**Authors:** Zafar Salman, Abdullah Muhammad, Dongil Han

**Affiliations:** Department of Computer Science and Engineering, Sejong University, Seoul, Republic of Korea

**Keywords:** deep learning, disease classification, computer vision, plant disease, mixture of experts (MoE), vision transformers

## Abstract

Plant disease classification using deep learning techniques has shown promising results, especially when models are trained on high-quality images. However, these models often suffer from a significant drop in their accuracies when tested in real-world agricultural settings. In the wild, models encounter images that are significantly different from the training data in aspects like lighting conditions, capturing conditions, image resolution, and the severity of disease. This discrepancy between the training images and images in-the-wild conditions poses a major challenge for deploying these models in agricultural settings. In this paper, we present a novel approach to address this issue by combining a Vision Transformer backbone with a Mixture of Experts, where multiple expert models are trained to specialize in different aspects of the input data, and a gating mechanism is implemented to select the most relevant experts for each input. The use of Mixture of Experts allows the model to dynamically allocate specialized experts to different types of input data, improving model performance across diverse image conditions. The approach significantly improves performance on diverse datasets that contain a range of image capturing conditions and disease severities. Furthermore, the model incorporates entropy regularization and orthogonal regularization, aiming to enhance the robustness and generalization capabilities. Experimental results demonstrate that the proposed model achieved a 20% improvement in accuracy compared to Vision Transformer (ViT). Furthermore, it demonstrated a 68% accuracy on cross-domain datasets like PlantVillage to PlantDoc, surpassing baseline models such as InceptionV3 and EfficientNet. This highlights the potential of our model for effective deployment in dynamic agricultural environments.

## 1 Introduction

In recent years, deep learning models like Convolutional Neural Networks (CNNs) have shown promising results for object detection and image classification. A number of models based on deep learning have been proposed, which cover a wide range of plant disease identification and classification (Salman et al., 2023). CNN-based methodologies have showcased remarkable accuracy in classifying various diseases across different crops (Mohanty et al., 2016). Renowned for their adeptness in capturing intricate visual features (Sladojevic et al., 2016), CNNs form the foundation of numerous object detection frameworks, featuring popular variants such as two-stage and one-stage detectors (Redmon et al., 2016). By employing multilayered convolutions, CNN architectures effectively extract object features and adjust parameters, which helps mitigate issues such as overfitting (Krizhevsky et al., 2012). Numerous deep learning models have been proposed for plant disease augmentation, identification, and classification, demonstrating remarkable accuracy across various crops (Muhammad et al., 2023). While two-stage detectors like Region-Based CNN (R-CNN) (Girshick et al., 2014) prioritize localization accuracy, they often compromise on speed, unlike one-stage detectors, which prioritize inference speed over precise localization.

Although significant progress has been made in computer vision and Artificial Intelligence (AI) for crop monitoring and disease detection, several critical challenges still persist. One key challenge is the accuracy gap between training datasets and real-world applications, as models trained on homogeneous data often struggle to generalize effectively in diverse, uncontrolled environments. Moreover, variations in leaf morphology, object size, and disease manifestations significantly impact model performance, while factors such as diverse lighting conditions, backgrounds, and object instances further intensify these challenges. These shortcomings highlight the need for robust models that are capable of adapting to dynamic environmental conditions for accurate disease detection.

The focus of this study is to address the challenge of bridging the performance gap between plant disease classification models trained on lab-controlled datasets and their deployment in such agricultural settings where image conditions vary from the training dataset. The proposed model in this study employs Vision Transformers (ViTs) for feature extraction and incorporates a Mixture of Experts (MoE) (Shazeer et al., 2017) architecture for decision-making, which enhances its adaptability and generalization capabilities. The addition of novel regularization techniques further enhances robustness while ensuring the balanced use of expert classifiers. Experimental results presented in this study show significant improvements in accuracy, demonstrating the potential of this approach for practical applications in plant disease detection.

## 2 Related work

### 2.1 Architectures

Recently, Transformer-based (Vaswani et al., 2017) methods have emerged in computer vision research and gained popularity due to their remarkable performance. Originally developed for Natural Language Processing (NLP) tasks, transformers revolutionized the field by introducing self attention mechanisms, enabling models to assign dynamic importance to different input elements. ViTs adapt this mechanism by treating image patches as input tokens, allowing global context modeling across the image. Transformers compute all pairwise interactions simultaneously, making them well-suited for modeling complex dependencies. ViTs implement this by splitting images into patches similar to word tokens. Although ViTs lack the inductive biases of CNNs—requiring more data for robust generalization—they excel at capturing global dependencies and have outperformed traditional CNNs like ResNet (He et al., 2016) in image classification tasks. This has led to the development of improved ViT architectures for computer vision.

**Figure 1:**
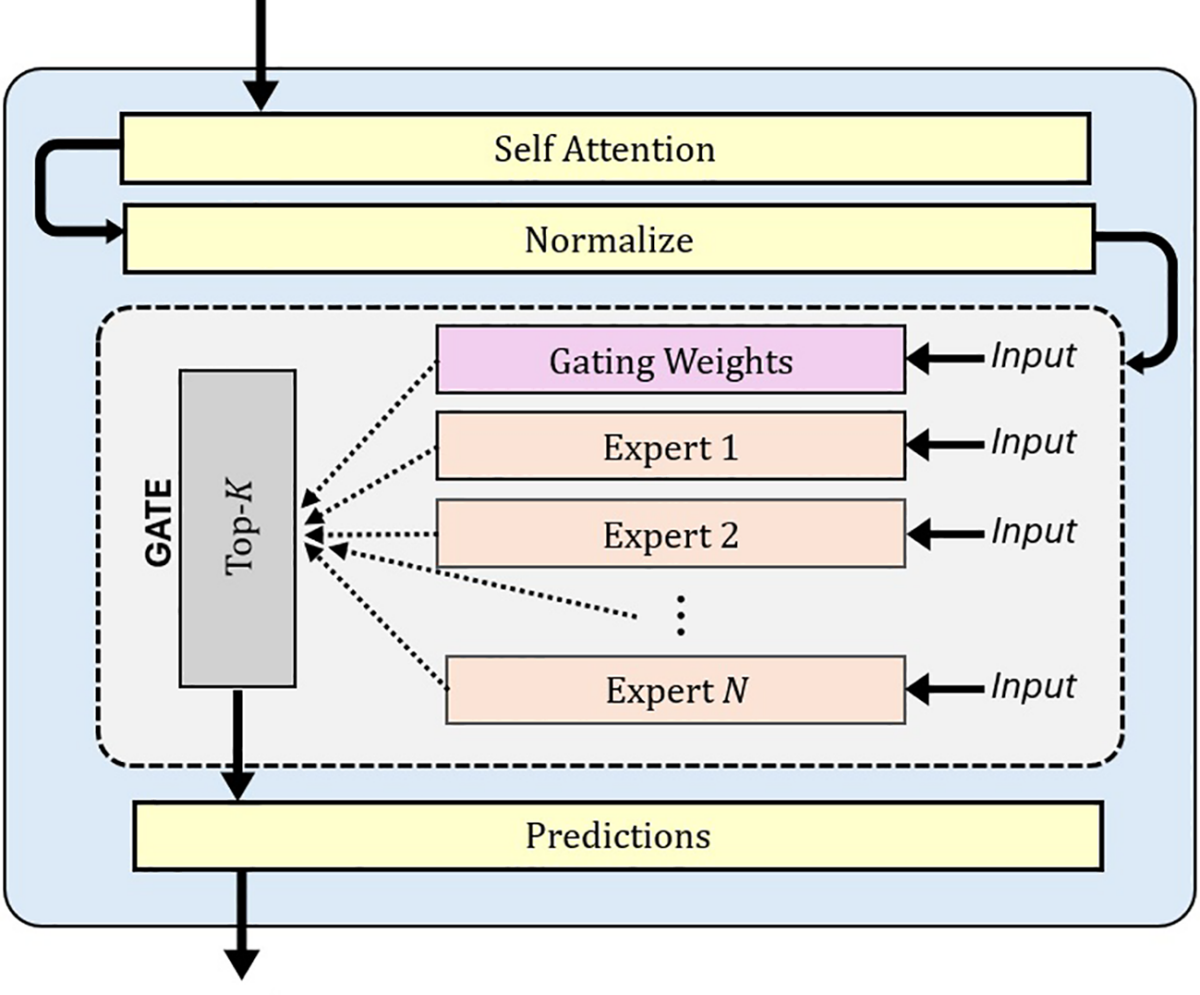
Diagram showing the integration of a MoE with a specialized gating mechanism that dynamically routes inputs through N expert networks. The gating weights determine which experts are activated for each input, enabling efficient specialization and adaptive processing. MoE, Mixture of Experts.

The MoE model (Shazeer et al., 2017) is an advanced machine learning framework that divides complex problems into simpler sub-tasks, each handled by specialized sub-networks called experts. MoE has demonstrated superior performance compared to models like LLaMA and GPT-3.5 (Jiang et al., 2023). A MoE model has two key components: expert networks and a gating mechanism. The experts consist of multiple lightweight Multilayer Perceptron (MLP) Layers. Each expert is trained to focus on specific aspects of a task (Huang et al., 2023). These experts are computationally efficient and scalable, allowing the model to grow in capacity with little increases in computational cost. A gating network, illustrated in **Figure 1**, dynamically selects the most relevant experts for each input. The final output is a weighted combination of these experts.

By implementing experts for separate tasks or data segments, MoE models benefit from a variety of perspectives and insights, boosting their overall performance. This flexibility not only enhances their ability to generalize but also enables the model to handle a wider range of challenges while maintaining both accuracy and efficiency.

### 2.2 Datasets

Numerous publicly available datasets have been developed for plant disease classification, reflecting the growing interest and research in this domain. Among these, the PlantVillage and PlantDoc datasets have been widely adopted in the literature due to their accessibility and broad usage across diverse studies. In this work, these two datasets are selected to evaluate the proposed model, as they provide a reliable benchmark for both controlled and real-world conditions in plant disease detection.

#### 2.2.1 PlantVillage

**Figure 2:**
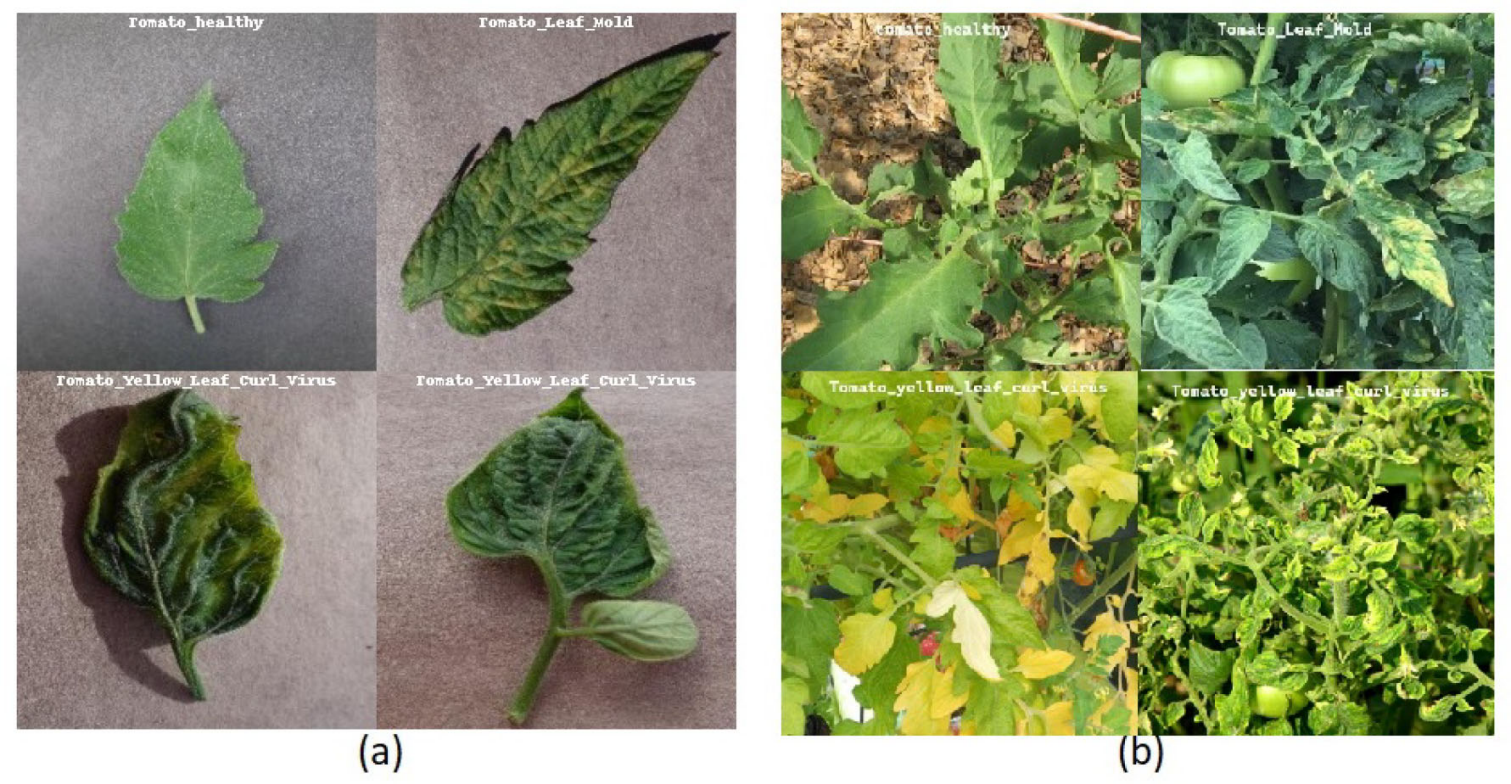
Challenges to in-the-wild classification **(a)** containing images of Tomato Healthy, Tomato Leaf Mold, and Tomato Yellow Leaf Curl Virus in PlantVillage dataset that are used for training. **(b)** Images of Tomato Healthy, Tomato Leaf Mold, and Tomato Yellow Leaf Curl Virus in PlantDoc dataset in the wild.

The Plant Village dataset (Hughes and Salathé, 2015) contains 54,306 images illustrating both healthy and infected leaves. The dataset contains a total of 38 classes, covering 14 different crop species and 26 distinct diseases. Due to its diverse representation of crops and disease conditions, the dataset has become a valuable resource for researchers and professionals in the field of plant disease detection (Ferentinos, 2018). It provides a robust foundation for training and evaluating machine learning models, thereby contributing to advancements in agricultural disease diagnosis and management. **Figure 2a** shows sample images of Tomato Healthy, Tomato Leaf Mold and Tomato Yellow Leaf Curl Virus from the PlantVillage dataset.

#### 2.2.2 PlantDoc

The PlantDoc dataset (Singh et al., 2020), introduced in 2019, consists of 2,598 images of both healthy and diseased plant leaves. The images were collected from various sources, including online platforms such as Google and Ecosia. This dataset encompasses 13 crop types affected by 17 different diseases. **Figure 2b** shows sample images of Tomato Healthy and Tomato Yellow Leaf Curl Virus from the PlantDoc dataset. A notable feature of PlantDoc is that it captures images under real-field conditions, offering a more realistic representation of the challenges faced by plant disease detection models in practical applications.

### 2.3 Challenges in plant disease classification

Many studies have employed identical datasets for both training and testing, resulting in high accuracy rates due to inherent dataset similarities. For instance, Mohanty et al. (2016) reported a remarkable 99.35% accuracy when their model, trained on the PlantVillage dataset (Hughes and Salathé, 2015), was tested within similar conditions. However, when subjected to in-the-wild images, their accuracy plummeted to below 40%. This drop in accuracy highlights the critical need for more comprehensive datasets and improved techniques to enhance model robustness and generalization to enable accurate disease detection.

**Figure 3:**
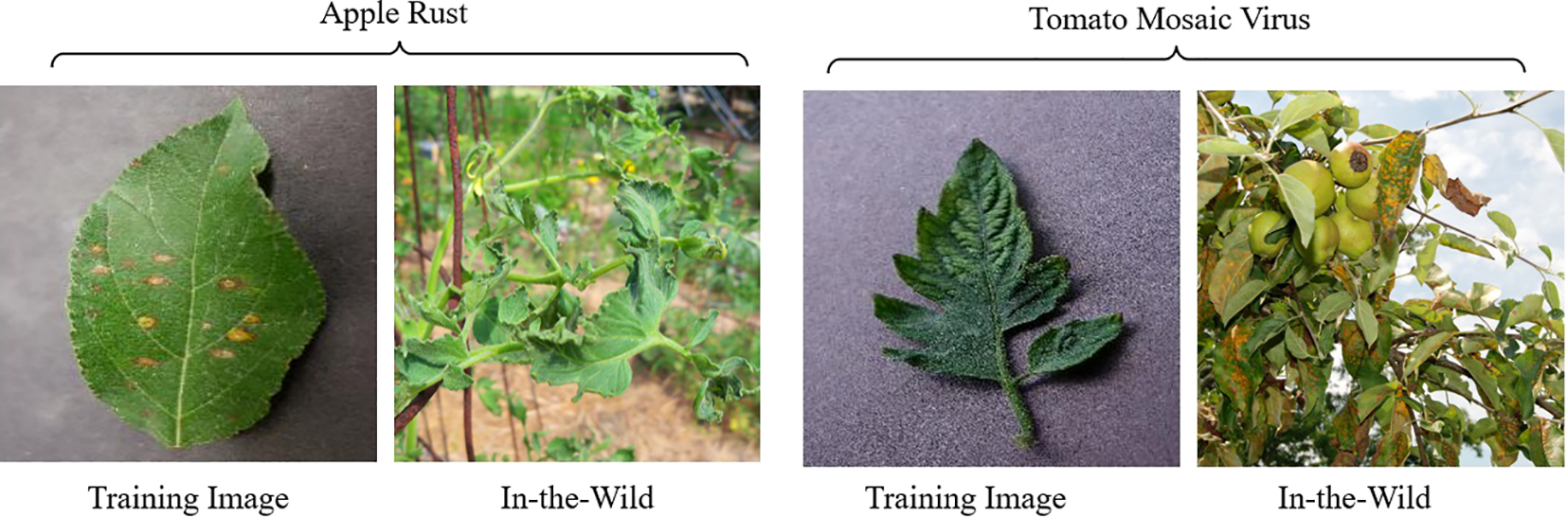
Comparison of images captured at different distances. The image taken from a greater distance shows a smaller object size and less clear leaf shapes, which may obscure diseased areas and complicate disease recognition tasks.

Object size also plays a crucial role in model performance, as it is closely affected by the distance between the camera and the object during image capture (Arnal Barbedo, 2013). Object sizes in images captured from greater distances tend to be smaller, which can hinder disease recognition tasks by obscuring diseased areas. Consequently, model performance suffers, as the diseased portions are not visible in those images, leading to diminished accuracy in disease identification and classification, as shown in **Figure 3**. To tackle these challenges effectively, it is crucial to develop image classification models that exhibit robustness and adaptability to the variation in the size of the diseased portion of a plant.

**Figure 4:**
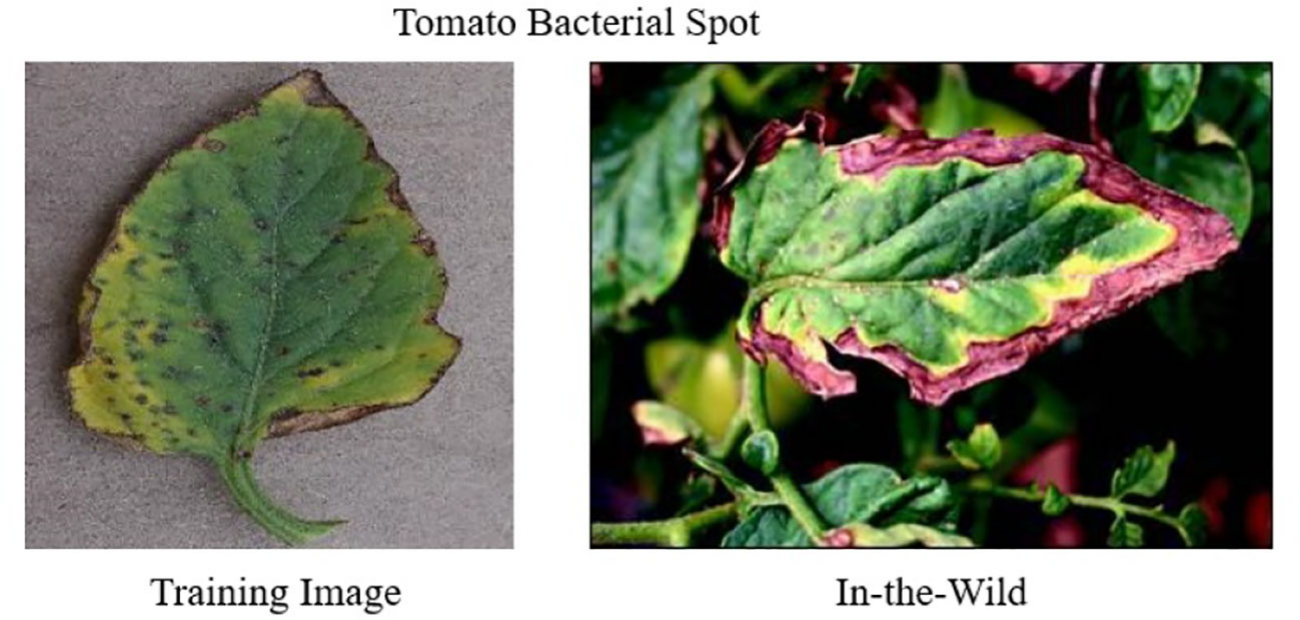
A Comparison of training (left) and testing image (right) samples. The training image displays early-stage disease severity, while the testing image, illustrating real-field encounters, depicts more advanced disease manifestations.

Moreover, the severity of the disease present in the training dataset plays a crucial role in model performance. The stage of the disease directly affects the accuracy of the model when applied in scenarios, as shown in **Figure 4**. Early-stage symptoms are usually subtle and harder to detect, whereas advanced stages present more evident symptoms, affecting the model’s ability to generalize across different stages of disease progression (Picon et al., 2019).

**Figure 5:**
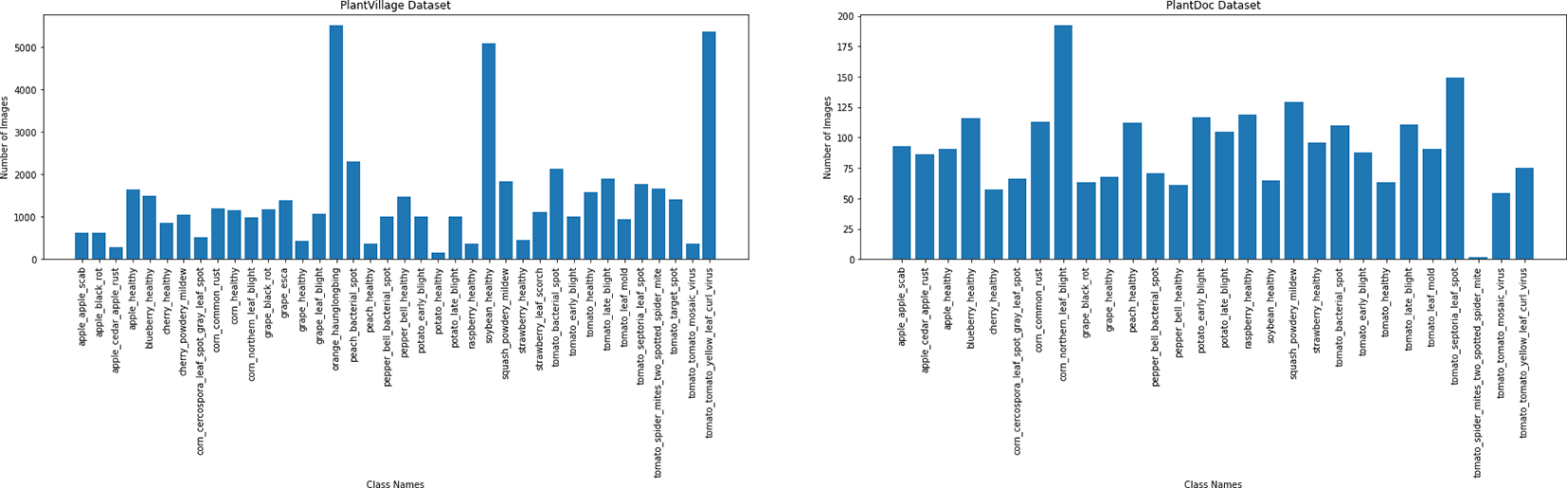
Image distribution in the PlantVillage (top) and PlantDoc (bottom) datasets. A significant class imbalance is observed in PlantVillage dataset, with tomato-related classes constituting 43.4% of the total images. This skewed distribution can lead to biased model performance, favoring overrepresented classes.

In addition to the need for larger datasets, it is important to highlight that datasets with detailed annotations and various stages of symptoms are particularly scarce. While current datasets are useful, they often fall short in terms of diversity and accuracy. The lack of variability hurts the performance of deep learning models in practical situations, where conditions such as background, lighting, and object instances are often quite different from one another, as shown in **Figure 2**. Furthermore, datasets such as PlantVillage suffer from issues such as class imbalance. As shown in **Figure 5**, tomato samples alone account for 43.4% of the total images, potentially biasing models toward overrepresented classes. The PlantDoc dataset also presents certain limitations. Due to the absence of sufficient domain expertise during annotation, some images are incorrectly labeled, which can degrade model performance. Moreover, several images in the PlantDoc dataset contain images that have multiple diseases in a single image, which affects the overall accuracy of image 6.

Addressing these challenges is crucial for improving the performance of deep learning models. Models trained on datasets with these limitations encounter difficulties in adapting to the complexities of practical applications, highlighting the need for robust training approaches. In this paper, we present a unique approach to cater to these challenges by enhancing the model’s adaptability and improving recognition accuracy.

## 3 Methodology

This section offers a detailed overview of the proposed architecture and techniques used in this research. It covers the architecture and mathematical foundations of the methods utilized for this research.

### 3.1 Our model

#### 3.1.1 Vision Transformer backbone

**Figure 6:**
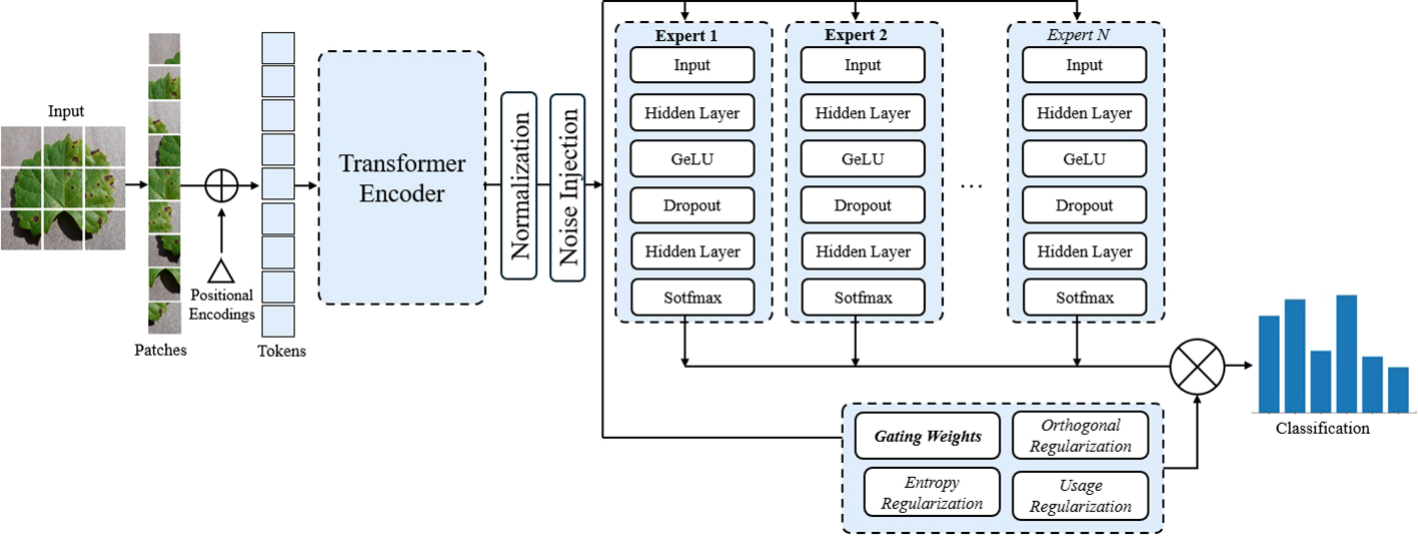
End-to-end architecture of the proposed ViT+MoE model for plant disease classification. Input images are split into patches and processed by a Vision Transformer encoder to extract global features. These features are normalized and routed through a Mixture of Experts (MoE) module, where a gating network assigns weights to multiple expert classifiers. Each expert contributes to the final prediction based on its assigned weight. Entropy, orthogonal, and usage regularizations are applied to ensure diverse, decorrelated, and balanced expert utilization. The final output is an aggregated classification score aimed at improving robustness. ViT, Vision Transformer.

Our proposed model, illustrated in **Figure 6**, employs a ViT encoder as the primary feature extractor. The motivation for using ViT as the backbone lies in its ability to capture long-range dependencies within image data. This allows the model to look at different parts of the image, making it effective for analyzing images with complex and changing conditions. Let **X** represent the input image. The ViT divides **X** into a sequence of image patches ${\left\{{\text{x}}_{i}\right\}}_{i=1}^{N}$ , where *N* denotes the total number of patches. Each patch **x** *_i_* is processed using a transformer-based architecture to capture long-range dependencies across the image. The output of the ViT can be denoted as a high-dimensional feature representation **H** ∈ *R^N^* ^×^ *^D^*, where *D* represents the feature dimension.

#### 3.1.2 Input normalization and noise injection

After feature extraction, the output **H** from the ViT is subjected to layer normalization, yielding $H_{\text{norm}}$ . This process stabilizes training by mitigating internal covariate shift, ensuring consistent gradient flow, and preventing exploding or vanishing gradients. It centers and scales feature distributions, enhancing convergence.

To improve generalization and prevent overfitting, Gaussian noise is added to *H*_norm_ during training. This regularization technique introduces variability, encouraging the model to learn robust features. The noise level, governed by the dataset characteristics, is kept low when critical features are subtle to avoid masking them, and higher when features are more prominent.

Formally, the perturbed features are as follows:


$$H_{\text{perturbed}}=H_{\text{norm}}+\epsilon ,\;\;{}^{\circ}\;\epsilon ∼N\left(0,\sigma^{2}\right)$$

where *σ* is a tunable hyperparameter controlling noise magnitude. Injecting zero-mean Gaussian noise *ϵ* into the normalized features enhances the model’s ability to generalize to unseen data.

#### 3.1.3 MoE layer

**Figure 7:**
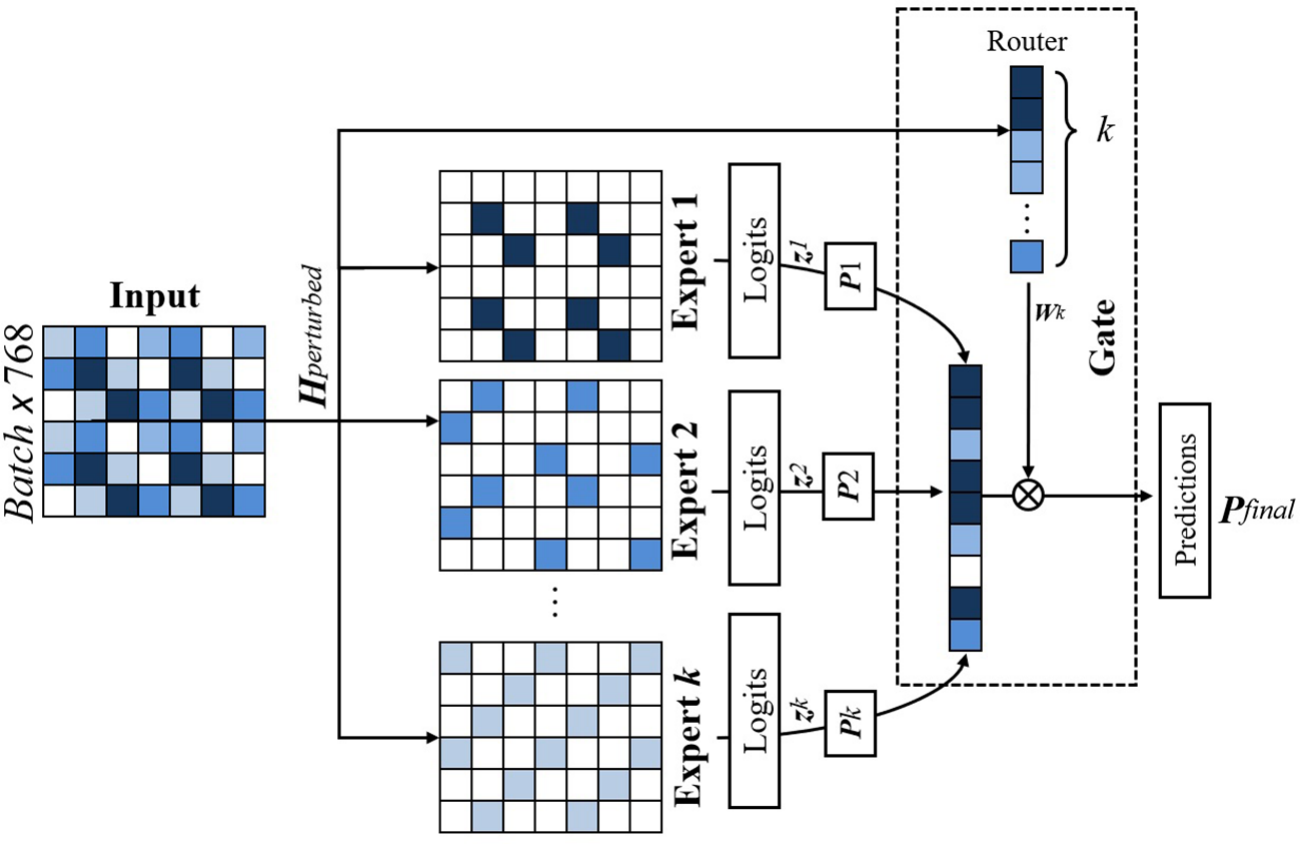
Figure illustrating gating mechanism of our proposed model. A gating network assigns weights to each expert based on the input, and the final prediction is obtained through a weighted sum of expert outputs. Entropy-based regularization ensures balanced expert contributions.

The core classification process is facilitated by a MoE Layer, comprising expert classifiers. The output of the MoE is a weighted sum of the selected individual expert predictions, with the weights determined by the gating mechanism. This process is illustrated in **Figure 7**.

##### 3.1.3.1 Expert classifiers

Each expert classifier within the MoE architecture consists of two fully connected layers, denoted as *W*_1_ and *W*_2_, interleaved with a dropout layer to mitigate overfitting. Let *z_k_* be the output of the *k*th expert after processing the input features. The final output of each expert is a softmax distribution *p_k_* = *softmax*(*z_k_*), representing the expert’s prediction probabilities across the target classes.

##### 3.1.3.2 Gating mechanism

The gating mechanism is modeled as a neural network *g*(·) that takes the normalized features $H_{norm}$ as input and outputs a set of weights *w* = *g*(*H_norm_*) ∈ *R^K^*, where *K* is the number of selected experts for the input. The weights *w* determine the contribution of each expert’s output *p_k_* to the final decision. The gating network is trained concurrently with the experts and includes an entropy-based regularization term to promote a balanced distribution of weights, thereby preventing any single expert from dominating the final prediction.

### 3.2 Training and loss functions

The model’s training process is guided by a combination of loss functions designed to ensure accurate predictions and robust generalization. The classification loss, denoted as *L_class_*, is computed using CrossEntropy Loss, which measures the difference between the predicted class probabilities and the true class labels. This loss drives the model to make accurate classifications.


$$L_{class}=-\frac{1}{N}\sum_{i=1}^{N}y_{i}\text{ log }({\hat{y}}_{i})$$

where

- *y_i_* is the true label for the *i*th instance.
-
${\hat{y}}_{i}$ is the predicted probability for the true class.
- *N* is the number of instances.

The gating loss $L_{gating}$ is applied to the output of the gating network. It is also a Cross-Entropy Loss that ensures the gating network assigns appropriate weights to each expert based on their specialization.


$$L_{gating}=-\frac{1}{N}\sum_{i=1}^{N}g_{i}\text{ log }({\hat{g}}_{i})$$

where

-
$g_{i}$ is the true gate target for the *i*th instance.
-
${\hat{g}}_{i}$ is the predicted gating probability for each expert.

A typical implementation of MoE can cause the over-reliance on a subset of experts, where the gating mechanism favors certain experts repeatedly due to their over-confidence, potentially disregarding inputs from other experts. This overconfidence could result in suboptimal performance, as the model underutilizes the full range of distinct features learned across all experts, potentially resulting in biased outputs. Hence, an Entropy Regularization Loss *L_entropy_* (Grandvalet and Bengio, 2004) is applied to the gating mechanism to address overconfidence. This loss penalizes low-entropy distributions in the gating outputs, encouraging a balanced contribution from all experts and preventing any single expert from dominating the predictions. It is given by the following:


$$L_{entropy}=-\frac{1}{N}\sum_{i=1}^{N}\sum_{j=1}^{K}g_{ij\;}\text{log }(g_{ij})$$

where

-
$g_{ij}$ is the gating weight for the *j*th expert on the *i*th instance.
- *K* is the total number of selected experts.

Employing multiple experts introduces the risk of redundancy, where several experts may end up learning similar features. This overlap can reduce the diversity of representations, ultimately leading to biased final decisions. To mitigate this, we incorporate orthogonal regularization (Bansal et al., 2018), which encourages each expert to learn distinct, non-overlapping features. This promotes specialization among experts and enhances overall model performance by ensuring more diverse and complementary representations. The Orthogonal Loss *L_orthogonal_* penalizes the similarity between the weight matrices of different experts by minimizing the Frobenius norm of their product. In doing so, it enforces diversity among the experts, thereby reducing redundancy and improving the performance of the ensemble. This is calculated as follows:


$$L_{orthogonal}=\sum_{i=1}^{K}\sum_{j=i+1}^{K}{∥W_{i}W_{j}^{⊤}∥}_{F}$$

where

- *W_i_* and *W_j_* are the weight matrices of the *i*th and *j*th experts, respectively.
-
${∥\cdot ∥}_{F}$ denotes the Frobenius norm.

While orthogonal and entropy regularization help balance expert selection and promote feature diversity, another challenge arises when certain experts are consistently assigned fewer inputs, limiting their contribution to the overall model capacity. To address this, we incorporate usage regularization (Steiner et al., 2021), which penalizes the under-utilization of experts and encourages the gating mechanism to distribute inputs more evenly. This ensures that all experts remain actively engaged and contribute meaningfully to the learning process. The corresponding usage regularization loss $L_{usage}$ complements the orthogonal and entropy losses, enabling the model to achieve a balance between accurate predictions, diverse expert contributions, and robust generalization. This loss is calculated as follows:


$$L_{usage}=\sum_{j=1}^{K}{\left(\frac{1}{N}\sum_{i=1}^{N}g_{ij}-\frac{1}{K}\right)}^{2}$$

The total loss $L_{total}$ is a weighted sum of the above losses:


$$L_{\text{total}}=L_{\text{class}}+L_{\text{gating}}+\lambda_{\text{entropy}}L_{\text{entropy}}+\lambda_{\text{orthogonal}}L_{\text{orthogonal}}+\lambda_{\text{usage}}L_{\text{usage}}$$

where

• $\lambda_{entropy}$ , $\lambda_{orthogonal}$ , and $\lambda_{usage}$ are the weights for the entropy regularization, orthogonal, and expert usage losses, respectively.

#### 3.2.1 Aggregation and prediction

The final prediction is produced by averaging the weighted outputs of the experts. This approach allows the model to make use of diverse perspectives provided by each expert while ensuring that the most relevant experts have a more significant influence on the final decision. Formally, the final prediction $p_{final}$ is computed by aggregating the outputs from all selected experts ${\left\{p_{k}\right\}}_{k=1}^{K}$ using the gating weights *w*. The aggregation process can be expressed as follows:


$$p_{final}=\sum_{k=1}^{K}w_{k}p_{k}$$

This weighted sum $p_{final}$ serves as the final prediction, combining the collective knowledge of the experts while emphasizing those most relevant according to the gating mechanism.

## 4 Experimental setup

### 4.1 Dataset analysis

In our analysis of the PlantVillage dataset, we identified key issues related to class imbalance and image redundancy. Certain classes were heavily overrepresented, while others contained very few samples, increasing the risk of biased predictions favoring dominant classes. Moreover, many images within a class showed minimal variation, limiting the model’s ability to generalize and effectively distinguish between disease symptoms.

To better understand the disparities between the PlantVillage and PlantDoc datasets, we performed a detailed comparison using multiple distributional and visual metrics. First, we computed the Kullback–Leibler (KL) divergence (Kullback and Leibler, 1951) using features from a pretrained ResNet50 (He et al., 2016) model. Class-wise KL divergence quantified feature distribution shifts between corresponding classes, while overall divergence provided a broader view of dataset disparity (see **Table 1**).

**Table 1:** Class-wise and overall KL divergence for PlantVillage and PlantDoc datasets using ResNet50 pretrained model.

Specie	Class/disease	KL divergence
Apple	Scab	0.1868
Apple	Rust	0.2503
Apple	Healthy	0.2059
Blueberry	Healthy	0.0750
Cherry	Healthy	0.3250
Corn	Cercospora Leaf Spot/Gray Leaf Spot	0.0280
Corn	Common Rust	0.1214
Corn	Northern Leaf Blight	0.3126
Grape	Black Rot	0.2008
Grape	Healthy	0.0916
Peach	Healthy	0.1618
Pepper bell	Healthy	0.2572
Potato	Early Blight	0.1556
Potato	Late Blight	0.1077
Raspberry	Healthy	0.2786
Soybean	Healthy	0.1165
Squash	Powdery Mildew	0.1227
Strawberry	Healthy	0.5049
Tomato	Bacterial Spot	0.1647
Tomato	Early_blight	0.1820
Tomato	Healthy	0.2526
Tomato	Late Blight	0.2903
Tomato	Leaf Mold	0.3465
Tomato	Septoria Leaf Spot	0.2072
Tomato	Spider Mites	1.3214
Tomato	Mosaic Virus	0.3889
Tomato	Yellow Leaf Curl Virus	0.0672
Overall		0.1883

KL, Kullback–Leibler.

**Figure 8:**
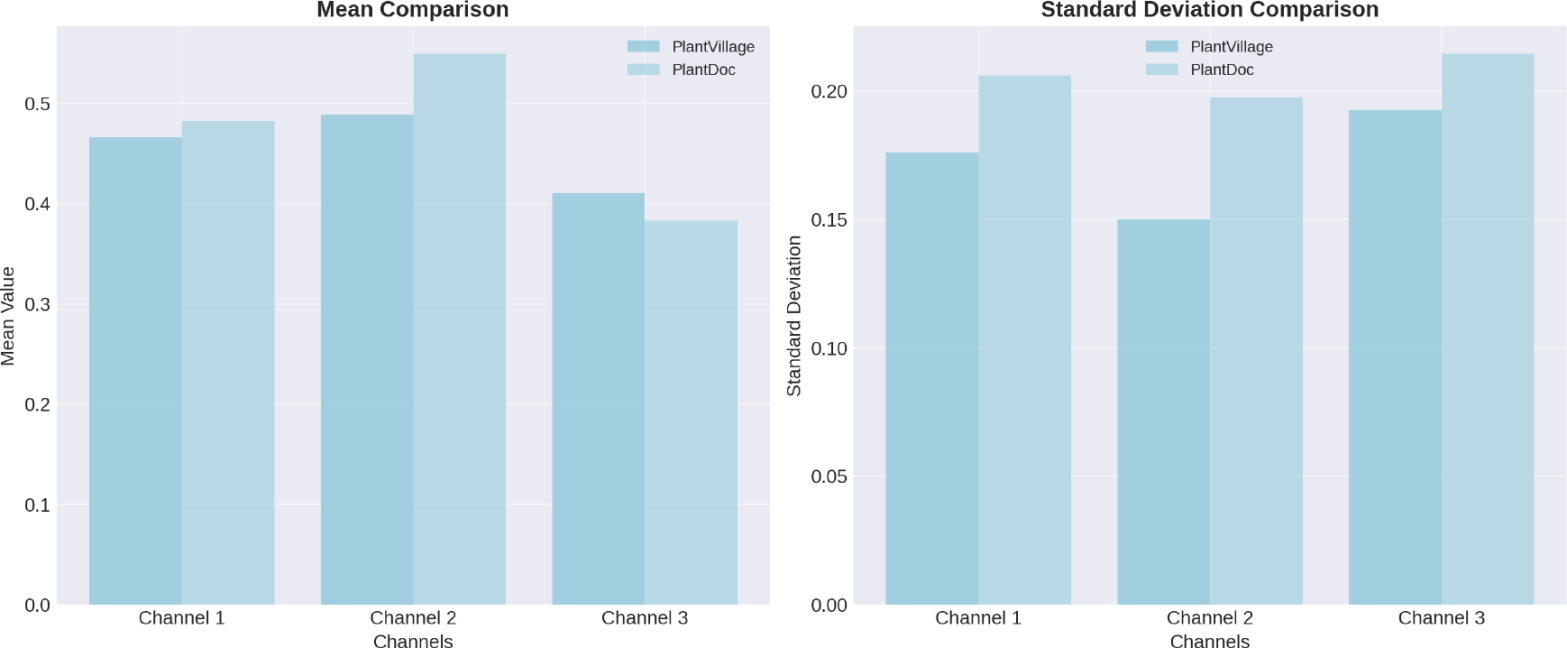
Analysis of pixel-level distributions reveals significant differences between the PlantVillage and PlantDoc datasets, highlighting the distinct characteristics and variability inherent in each dataset.

We further analyzed pixel-level distributions, comparing the mean and standard deviation of RGB channels across both datasets. As shown in **Figure 8**, the PlantDoc images exhibited higher mean intensity in the green and blue channels, as well as increased standard deviation, suggesting greater brightness and variability in lighting and color conditions.

These differences go beyond visual appearance, reflecting the diversity of real-world scenarios that the model may encounter. Training on PlantVillage and testing on PlantDoc provides a robust evaluation of the model’s ability to generalize to new environments beyond the training distribution.

### 4.2 Dataset preparation

**Figure 9:**
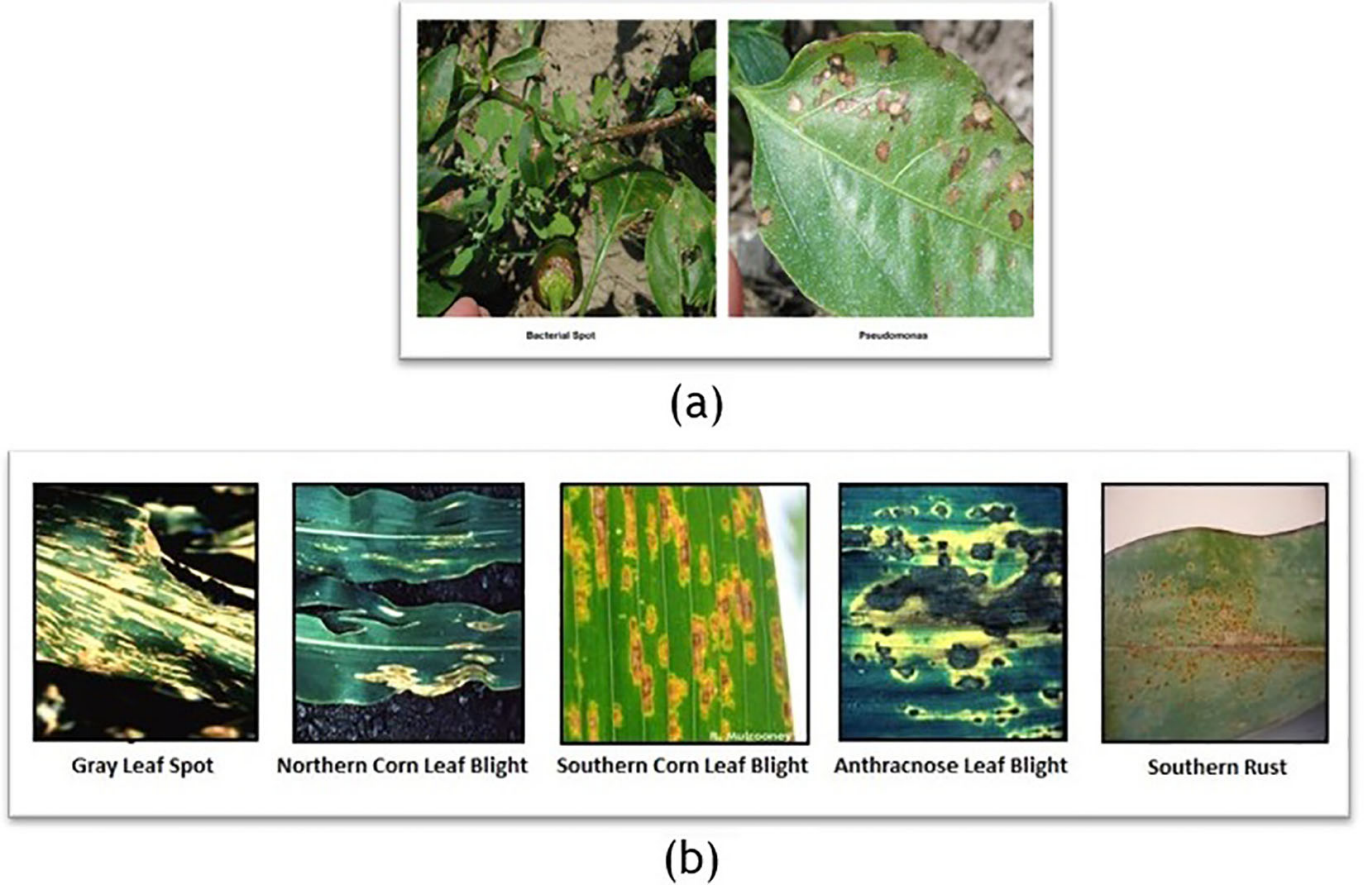
Examples from the PlantDoc dataset illustrating annotation and structural challenges. **(a)** An image exhibiting examples of both bacterial spot and pseudomonas, yet labeled as a single disease, which introduces ambiguity during training and evaluation. **(b)** A composite image containing five distinct subimages, each depicting different diseases, but collectively treated as a single data point. Such cases can mislead the model during learning by conflating multiple disease features, thereby reducing classification accuracy and reliability.

We addressed challenges like images showing multiple disease symptoms or irrelevant content (see **Figure 9**) in the PlantDoc Dataset. We split such composite images by symptom and removed unrelated images (e.g., fruits) to ensure that only relevant images were used as test input for the resulting classification.

**Figure 10:**
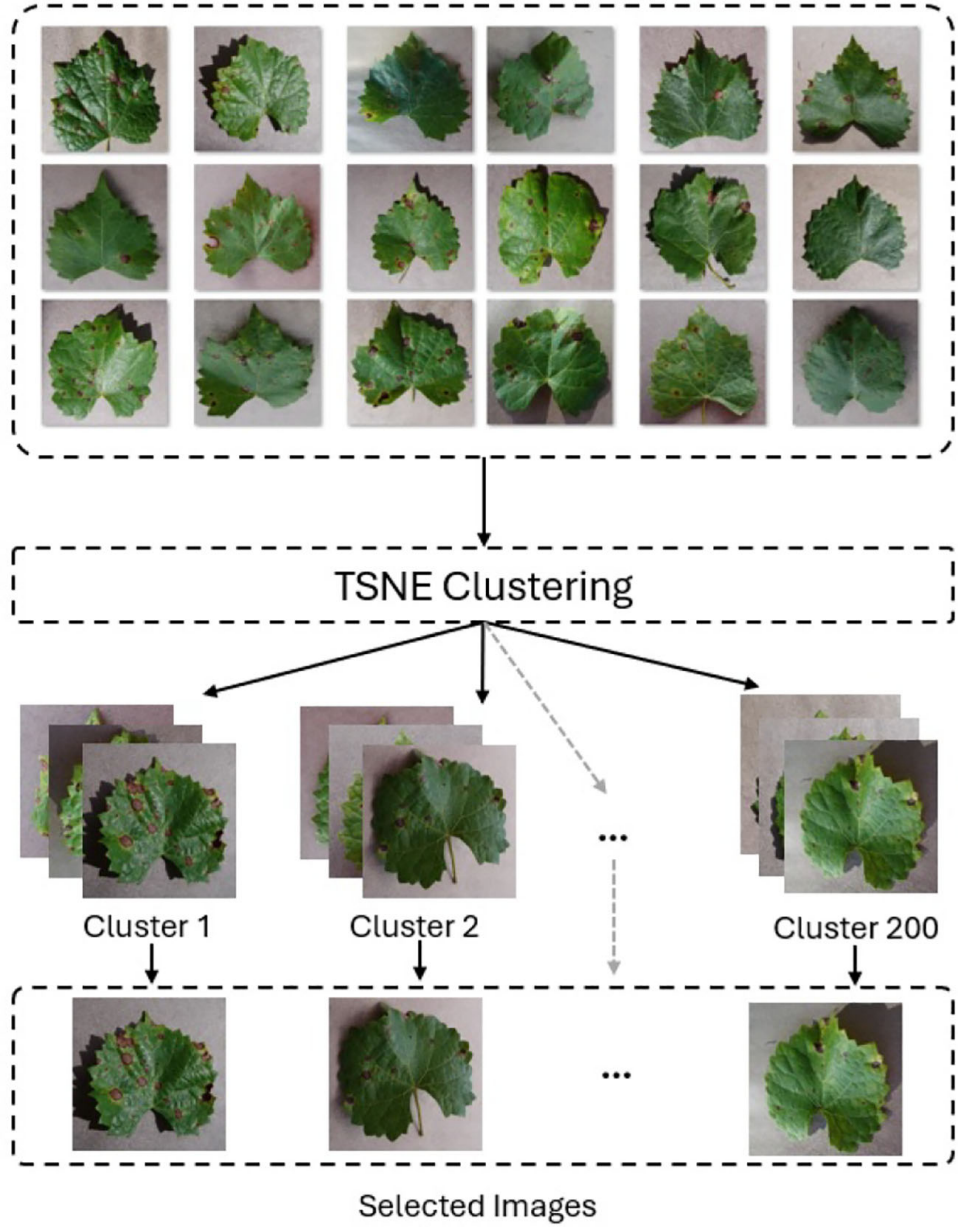
Downsampling using T-SNE clustering and VGG16 to extract 200 images per class in the PlantVillage dataset. One representative image is selected from each cluster to reduce redundancy.

To mitigate class imbalance in the PlantVillage dataset, we employed a downsampling strategy using t-distributed Stochastic Neighbour Embedding (T-SNE) clustering (Van der Maaten and Hinton, 2008) with VGG16 (Simonyan and Zisserman, 2014) for feature extraction. We created two balanced subsets, PlantVillage_100 and PlantVillage_200, by generating 100 and 200 clusters per class and selecting one representative image from each (see **Figure 10**). This method preserved class diversity while reducing redundancy and dataset size, enabling us to evaluate model robustness and generalization with limited data.

Feature vectors were extracted from the second-to-last fully connected layer of a pretrained VGG16 model, which was modified by removing the last three layers. Images were resized to 224 × 224 and normalized using standard ImageNet statistics. T-SNE was applied with two components, Euclidean distance, perplexity of 30, and learning rate of 500 to project features into a 2D space.

While clustering reduced repetition, it could risk excluding rare variations. To counter this, we prioritized representative images with clear disease symptoms regardless of severity. We selected these images manually. The goal was to balance dataset compactness with informative diversity, minimizing overfitting while retaining critical features for effective learning.

**Figure 11:**
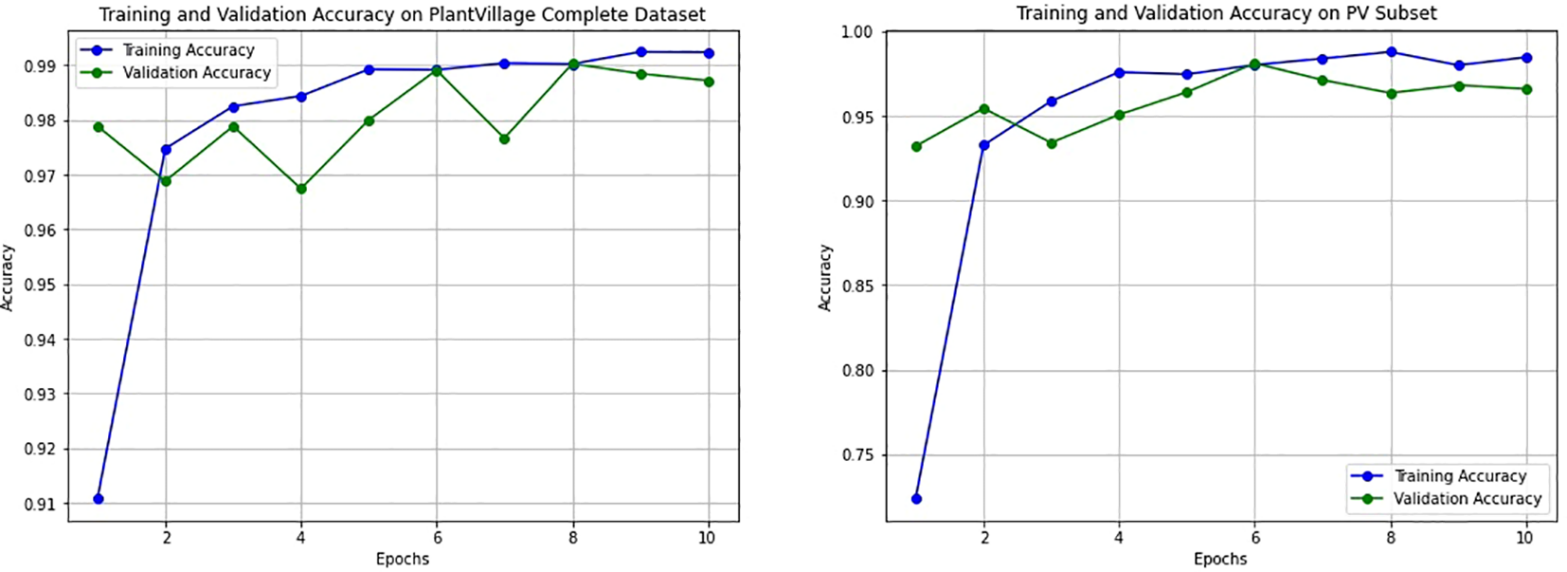
Training and validation accuracy curves for the VGG16 model trained on two datasets. The left plot shows performance on the full PlantVillage dataset (Model A), while the right plot corresponds to training on the downsampled PlantVillage 200 dataset (Model B). Both models exhibit smooth convergence with minimal overfitting, and Model B demonstrates stable learning behavior despite significantly reduced training data.

**Figure 12:**
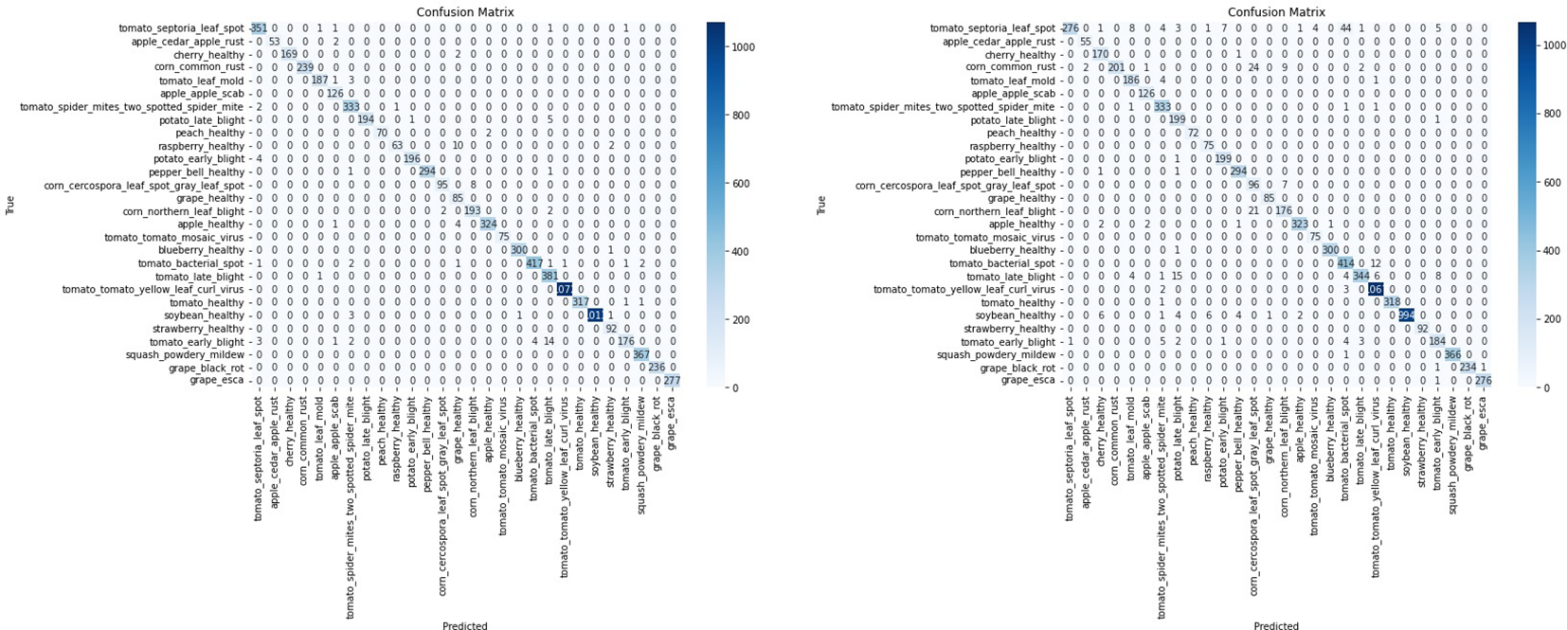
Confusion matrices illustrating classification performance of the VGG16 model on a shared test set. The left matrix represents the model trained on the complete PlantVillage dataset (Model A), while the right matrix corresponds to the model trained on the downsampled subset with 200 images (Model B). Despite the data reduction, Model B maintains competitive performance across most classes, indicating that the T-SNE-based downsampling preserves essential class-discriminative features.

To assess the impact of the T-SNE-based downsampling strategy on model performance, a performance drop analysis was conducted by training two VGG16 models— Model A on the full dataset and Model B on the downsampled subset. Both models were evaluated using a shared test set, and it was ensured that the images in the test set were not part of either model’s training set to prevent data leakage and ensure fair evaluation. The analysis revealed only a minor and negligible decrease in overall accuracy and F1-scores for the model trained on the downsampled data (**Figure 11**). As shown in **Table 2**, Model A achieved an accuracy and F1-score of 0.9872, while Model B, trained on a significantly smaller dataset (200 images), still reached a strong accuracy of 0.9660 and an F1-score of 0.9660. The minimal performance drop (2%) indicates that the T-SNE-based downsampling preserved essential data characteristics despite the drastic reduction in dataset size. Further analysis using the confusion matrices (**Figure 12**) supports these findings. While Model A demonstrates near-perfect classification across almost all classes, Model B exhibits only slight degradations. These results suggest that our downsampling strategy maintains the representativeness of the original dataset while significantly reducing data size.

**Table 2:** Comparison of validation metrics for two VGG16 models: Model A trained on the full dataset, and Model B trained on a T-SNE-based downsampled subset.

Model	Dataset	Accuracy	Precision	Recall	F1-score
Model A	PlantVillage	0.9872	0.9877	0.9872	0.9872
Model B	PlantVillage with 200 images	0.9660	0.9689	0.9660	0.9660

Both models were evaluated on a shared, unseen test set to ensure fair assessment. The minimal drop in accuracy and F1-score observed in Model B indicates that the downsampling strategy effectively preserved key data characteristics while significantly reducing dataset size.

### 4.3 Results and comparison

In this section, we present the results of experiments conducted to evaluate the performance of the proposed model. We compare these results against state-of-the-art architectures, including Vision Transformer (ViT-Base), InceptionV3 (Szegedy et al., 2015), EfficientNet (Tan and Le, 2019), and fine-grained classifiers such as Api-Net (Zhuang et al., 2020), CAL (Rao et al., 2021), and TransFG (He et al., 2022).

For architectural comparison, an ensemble CNN comprising DenseNet161 (Huang et al., 2017), ResNet152, and VGG19 was also implemented. Each base model was trained independently, and predictions were aggregated using majority voting to improve generalization and reduce variance. All models were evaluated across multiple datasets and data splits using metrics such as accuracy, F1-score, precision, and recall.

While recent research implementing deep learning models for plant identification and disease classification has predominantly relied on CNNs (Kamilaris and Prenafeta-Boldú, 2018), which are well-suited for visual analysis tasks, our study deliberately selected the most prominent CNN architectures to ensure a non-redundant comparison.

Our first set of experiments was conducted using an 80:10:10 data split, representing training, validation, and testing sets, on the PlantVillage dataset. These experiments included lab-controlled datasets for evaluating the baseline performance of our model and comparing it with other models. As shown in **Table 3**, our proposed model achieves an accuracy of 0.9996, slightly superior to Ensemble, ViT-Base, and other models. On PlantVillage, the performance differences between the models were marginal, demonstrating the proposed model’s reliability in well-controlled scenarios.

**Table 3:** Performance comparison of models using an 80:10:10 data split for training, validation, and testing.

Model	Dataset	Accuracy	F1-score	Precision	Recall
Ours	PlantVillage	0.9996	0.9996	0.9996	0.9996
Ensemble	PlantVillage	0.9996	0.9995	0.9995	0.9995
ViT-Base	PlantVillage	0.9995	0.9995	0.9995	0.9995
MoE	PlantVillage	0.9984	0.9984	0.9984	0.9984
InceptionV3	PlantVillage	0.9922	0.9888	0.9897	0.9922
EfficientNet	PlantVillage	0.9922	0.99	0.9894	0.9934
CAL	PlantVillage	0.9993	0.9993	0.9993	0.9993
API-Net	PlantVillage	0.9993	0.9993	0.9993	0.9993
TransFG	PlantVillage	0.9995	0.9995	0.9995	0.9995
Model	Data subset	Accuracy	F1-score	Precision	Recall
Ours	PlantDoc	0.74	0.74	0.77	0.74
ViT-Base	PlantDoc	0.69	0.60	0.61	0.59
API-Net	PlantDoc	0.69	0.68	0.69	0.69
Ensemble	PlantDoc	0.68	0.68	0.69	0.69
TransFG	PlantDoc	0.68	0.67	0.68	0.68
CAL	PlantDoc	0.68	0.67	0.67	0.66
InceptionV3	PlantDoc	0.65	0.64	0.67	0.65
EfficientNet	PlantDoc	0.59	0.55	0.53	0.59

However, a significant divergence in performance was observed when these models were tested on the PlantDoc sub-dataset, which consists of more challenging in-the-wild images. In these experiments, the proposed model exhibited remarkable robustness, achieving an accuracy of 0.74, outperforming all other models.

This superior performance highlights the generalization capabilities of the proposed model in handling diverse environmental conditions, such as varying lighting, backgrounds, and object scales. Compared to fine-grained classification models like CAL, API-Net, and TransFG, our model achieved higher accuracy, highlighting its effectiveness in complex scenarios.

To evaluate cross-domain generalization capabilities, the proposed model was trained on the PV_200 dataset, a balanced subset of PlantVillage, and tested on the PlantDoc sub-dataset. The results, presented in **Table 4**, show that the proposed model achieved an accuracy of 68%, which is significantly higher than the accuracies achieved by ViT-Base (48%), InceptionV3 (41%), and EfficientNet (43%). This demonstrates the model’s ability to bridge the gap between lab-controlled datasets and complex image conditions, thereby addressing one of the primary challenges in plant disease classification.

**Table 4:** Performance comparison of different models trained on PV_200 dataset.

Model	Training	In the wild	Accuracy	F1-score	Precision	Recall
Our model	PV_200	PlantDoc	0.68	0.65	0.67	0.64
MoE	PV_200	PlantDoc	0.60	0.57	0.56	0.60
Ensemble	PV_200	PlantDoc	0.51	0.50	0.50	0.50
ViT-Base	PV_200	PlantDoc	0.48	0.47	0.46	0.48
InceptionV3	PV_200	PlantDoc	0.41	0.40	0.40	0.40
EfficientNet	PV_200	PlantDoc	0.43	0.42	0.42	0.43
CAL	PV_200	PlantDoc	0.44	0.44	0.44	0.44
API-Net	PV_200	PlantDoc	0.39	0.38	0.38	0.39
TransFG	PV_200	PlantDoc	0.44	0.42	0.42	0.43

Further experiments were conducted to assess the model’s performance under limited data scenarios by training it on the PV_100 dataset, which contains only 100 images per class. Despite the reduced data availability, the proposed model maintained competitive performance, achieving an accuracy of 49% on PlantDoc, as shown in **Table 5**. This result demonstrates the robustness of the model and its adaptability to data-constrained settings, a critical factor for practical deployment where collecting large annotated datasets is often impractical.

**Table 5:** Performance comparison of different models trained on PV_100 sub-dataset.

Model	Training	In the wild	Accuracy	F1-score	Precision	Recall
Our model	PV_100	PlantDoc	0.49	0.51	0.53	0.49
Ensemble	PV_100	PlantDoc	0.41	0.40	0.44	0.36
ViT-Base	PV_100	PlantDoc	0.30	0.35	0.44	0.30
InceptionV3	PV_100	PlantDoc	0.29	0.29	0.28	0.29
EfficientNet	PV_100	PlantDoc	0.28	0.28	0.19	0.23
CAL	PV_100	PlantDoc	0.37	0.37	0.37	0.37
API-Net	PV_100	PlantDoc	0.32	0.32	0.32	0.32
TransFG	PV_100	PlantDoc	0.37	0.36	0.37	0.35

From the results presented in **Tables 3**–**5**, we can conclude that the ViT-MoE, coupled with entropy, orthogonal, and usage regularization, significantly enhances the model’s ability to generalize across domains and datasets. The proposed model not only excelled in controlled settings but also consistently outperformed other models in challenging scenarios, establishing itself as a robust and efficient solution for plant disease classification.

## 5 Discussion and ablation studies

Plant diseases vary significantly in their manifestation, with differences in shape, color, texture, and spatial spread. For example, some diseases manifest as distinct spots, such as target spot on tomato leaves and septoria leaf spot, while others cause widespread discoloration, like two-spotted spider mites and yellow leaf curl virus. Some diseases, like powdery mildew on squash, appear as powdery textures. A single model often struggles to capture all such nuances. The MoE allows multiple experts to specialize in different features such as shapes, colors, or textures, enabling the model to handle these diverse characteristics effectively. Similarly, ensemble methods combine the predictions of multiple diverse models to improve generalization by reducing variance and mitigating overfitting. Structurally, both MoE and ensemble approaches leverage multiple specialized components, making them particularly effective in handling complex and varied input data. As observed in our experiments, both methods demonstrated strong generalization capabilities, with improved accuracy stemming from their ability to compensate for the limitations of individual models through collaborative decision-making.

To further evaluate the impact of various components in our proposed model, we conducted an ablation study. This study systematically isolates and modifies key elements of our model to assess their contribution to overall performance. We examine the effect of the number of experts, entropy regularization, and orthogonal regularization on classification accuracy.

### 5.1 Effect of number of experts

**Figure 13:**
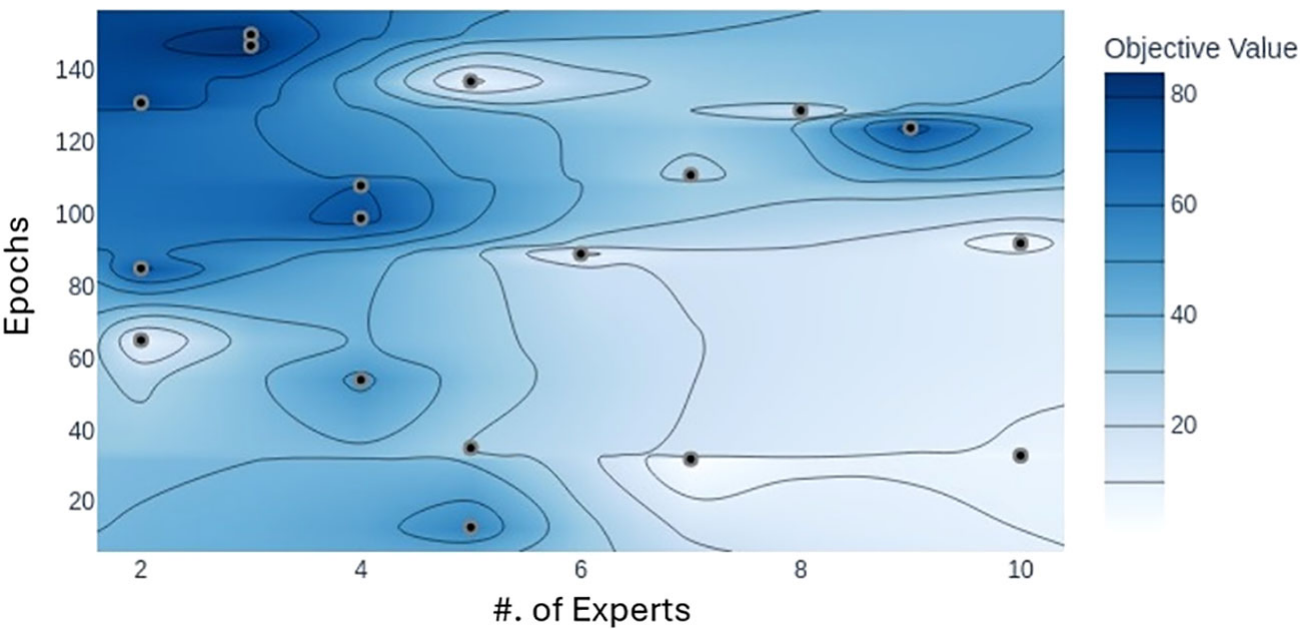
Plot illustrating the accuracy achieved with different numbers of experts with respect to epochs. Darker area represents the values closer to the maximum accuracy. Model achieves maximum accuracy when the total number of experts is three.

We utilized the MoE technique to improve the model’s generalization across varying image conditions. To assess its impact, we experimented by adjusting the number of selected experts in the model, testing configurations with top-*K* set between 2 and 10 for a total number of 10 experts on the dataset. Our findings indicate that the optimal number of experts in a MoE model depends on the need to capture fine-grained features for classification. It is very crucial for the model to have an appropriate number of experts with appropriate depth to establish a balance for optimizing the trade-off between overfitting and underfitting. When subtle variations in the data are critical for classification, increasing the number of experts can boost performance. However, adding too many experts does not necessarily improve accuracy, underscoring the importance of trial and error to find the best setup. This behavior is also depicted in **Figure 13**. Since each expert learns distinct features that collectively influence classification accuracy, we found that for our dataset, the optimal results were achieved with top-*K* set to 3.

### 5.2 Effect of entropy regularization

**Figure 14:**
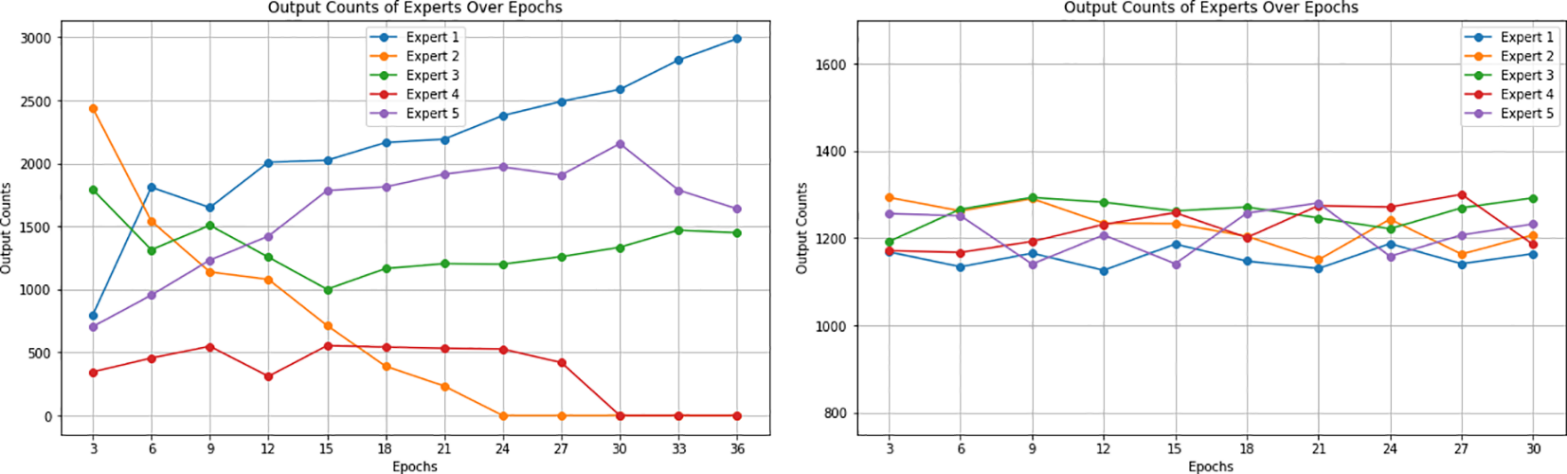
(Left) Contribution of five experts over epochs showing a decline for some experts after some epochs during training without entropy regularization. Figure shows some experts reaching zero contribution to output, while others dominate the output results. (Right) Contribution of five experts over epochs showing a balanced performance after the implementation of entropy regularization. The figure illustrates a more stable distribution of contributions, with all experts actively participating in the predictions.

Entropy regularization is incorporated to reduce model overconfidence. During the initial phases of model development, it was observed that with the training of the model, some experts started dominating the output results while ignoring the outputs of a subset of other experts due to their very high confidence scores. This behavior is shown in **Figure 14**. The entropy regularization ensures that no experts dominate the decision-making process. While this encourages models to consider balanced decision-making, it may lead to slower convergence during training, as the model balances between exploring various outputs and optimizing for accuracy. Hence, we implemented it as a hyperparameter to ensure that its balanced contribution leads to a better overall performance. To evaluate its effectiveness, we trained our model with various settings of entropy regularization. The results showed that high values of entropy regularization generally led to better model accuracy, indicating that modest entropy regularization can effectively mitigate overconfidence without significantly compromising performance.

### 5.3 Effect of orthogonal regularization

Orthogonal regularization promotes the independence of model parameters by encouraging them to be orthogonal to one another. When implemented as a loss function, it adds a penalty on the measure of deviation of weight vectors from being orthogonal. The goal of this regulation is to ensure that the features learned by the experts do not overlap too much. Hence, enforcing the experts to learn distinct features on the provided dataset during training leads to an informative aggregate output. This results in robustness against overfitting and redundancy. However, it comes at the cost of significant computational overhead while training, as the model needs to compute and apply the orthogonality constraints.

**Figure 15:**
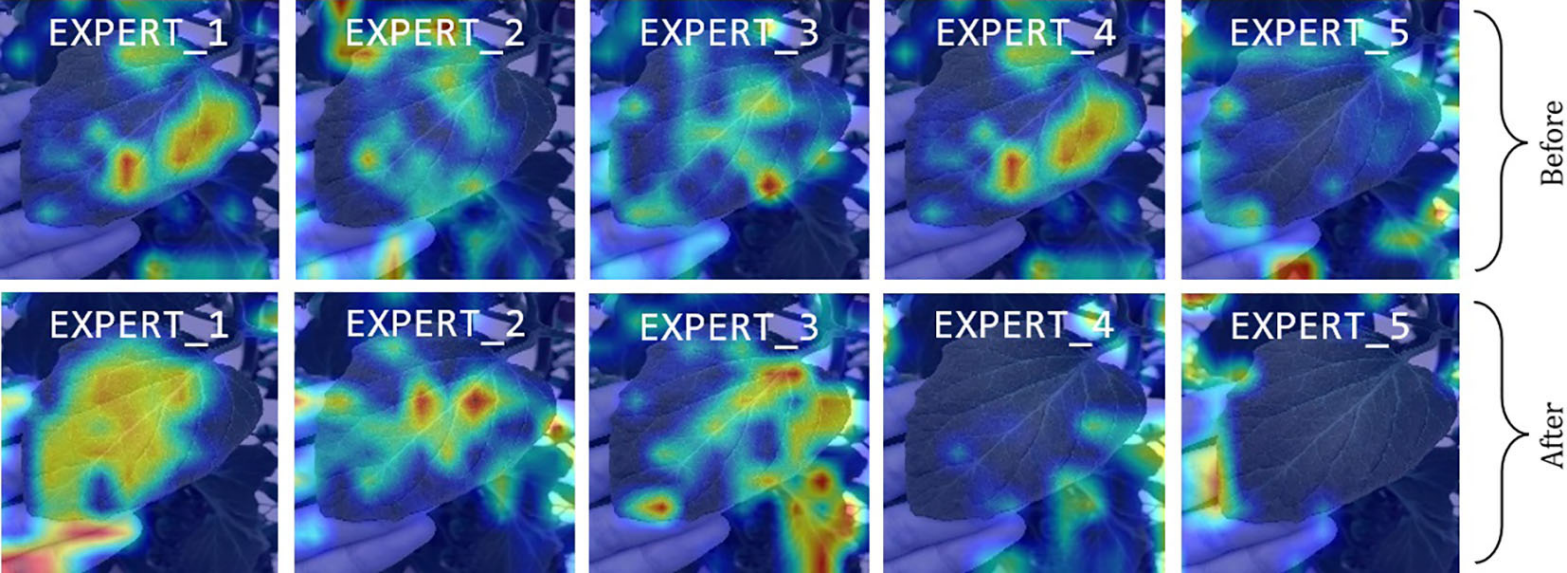
Visualization of experts’ activation maps in CNN–MoE study. The top row shows activation maps before orthogonal regularization, and the bottom row after. Expert 1 and expert 4 show similar activation maps. The bottom row highlights how orthogonal regularization promotes distinct, non-overlapping features. Red regions indicate higher activation regions, while blue represents lower activation areas. CNN, Convolutional Neural Network; MoE, Mixture of Experts.

To assess the effectiveness of orthogonal regularization, we performed an ablation study using a CNN backbone instead of ViT-B16. While our primary model utilizes ViT outputs into the MoE classifier, inspecting and visualizing expert activations directly from transformer outputs is challenging due to the high-dimensional nature of the embeddings and the lack of explicit spatial features. To overcome this, we employed a CNN-based MoE structure, which allowed for the visualization and analysis of expert activation maps for visualization purposes, as the key insights on the effect of orthogonal regularization are still relevant to both the CNN and ViT architectures. We trained this CNN-MoE model for 80 epochs on the PlantVillage dataset, which yielded an accuracy of 99%. **Figure 15** illustrates the activation maps of each expert before and after applying orthogonal regularization, showing how each expert learns to focus on different discriminative input regions. Prior to orthogonality regularization, expert 1 and expert 4 produce similar activation maps, but after implementing regularization, all experts learn distinct features.

### 5.4 Effect of usage regularization

Usage regularization aims to ensure that all expert classifiers contribute to the decision-making process. This technique is essential for mitigating the risk of overfitting to specific features learned by only a few experts. When a gate provides a high-confidence, unreliable prediction, the aggregated output is stabilized by contributions from other experts. This also comes with a trade-off; i.e., some of the experts are trained to capture such patterns that other experts are unable to capture due to the implementation of orthogonal regularization. Hence, it becomes very crucial to select an optimal weight for the usage regularization that balances the impact and does not override the impact of other regularization techniques.

### 5.5 Combined effect of regularization techniques

**Figure 16:**
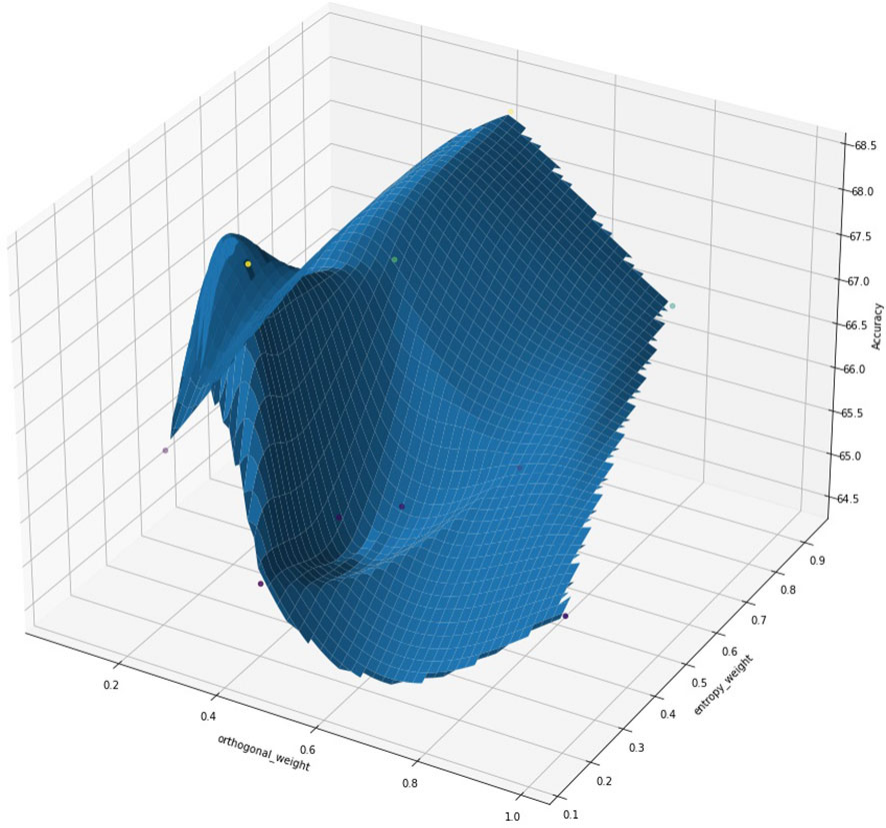
3D visualization of model accuracy as a function of orthogonal regularization weight and entropy regularization weight. The surface plot illustrates the complex interplay between these two regularization techniques and their impact on model performance. The red star indicates the point of maximum accuracy achieved.

To further understand the interplay between entropy and orthogonal regularization, we conducted a series of experiments varying both parameters simultaneously. **Figure 16** presents a 3D visualization of the model’s accuracy as a function of entropy and orthogonal regularization weights. The plot illustrates the relationship between these regularization techniques and model performance. We observe that the highest accuracy is achieved with a specific combination of moderate entropy and orthogonal regularization weights, indicated by the peak in the 3D surface. This visualization highlights the importance of carefully tuning these hyperparameters together, as their optimal values are interdependent. The plot also highlights the sensitivity of the model’s performance to these regularization techniques, with sharp drops in accuracy when the weights deviate significantly from their optimal values.

**Figure 17:**
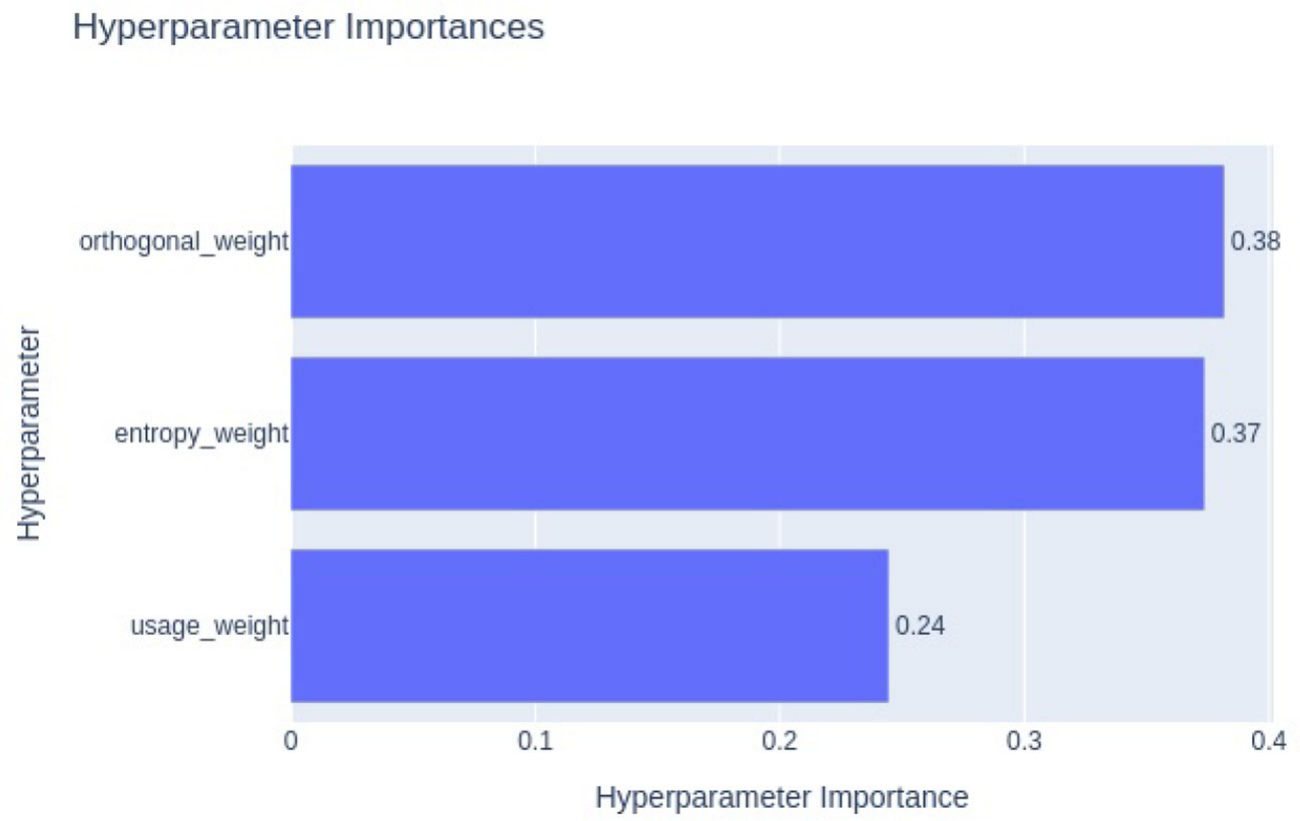
Optuna hyperparameter importance plot showing the relative impact of entropy, orthogonal, and usage regularization weights on validation accuracy. Entropy regularization shows the highest influence, followed by orthogonal and usage regularization.

To assess the contribution of each regularization term quantitatively, we conducted a hyperparameter optimization study using Optuna. Optuna’s hyperparameter importance analysis is based on model-agnostic techniques such as impurity-based feature importance, which estimate the relative influence of each hyperparameter on the objective value, which in our case is validation accuracy. The resulting importance plot, shown in **Figure 17**, ranks the regularization weights by their impact. The entropy regularization weight emerged as the most influential (importance = 0.38), followed by orthogonal regularization (0.37) and usage regularization (0.24). This indicates that entropy and orthogonal regularization play a more critical role in improving model generalization and gating behavior, consistent with our qualitative observations. The lower importance of usage regularization suggests that while it contributes to balanced expert activation, its role is more complementary.

**Figure 18:**
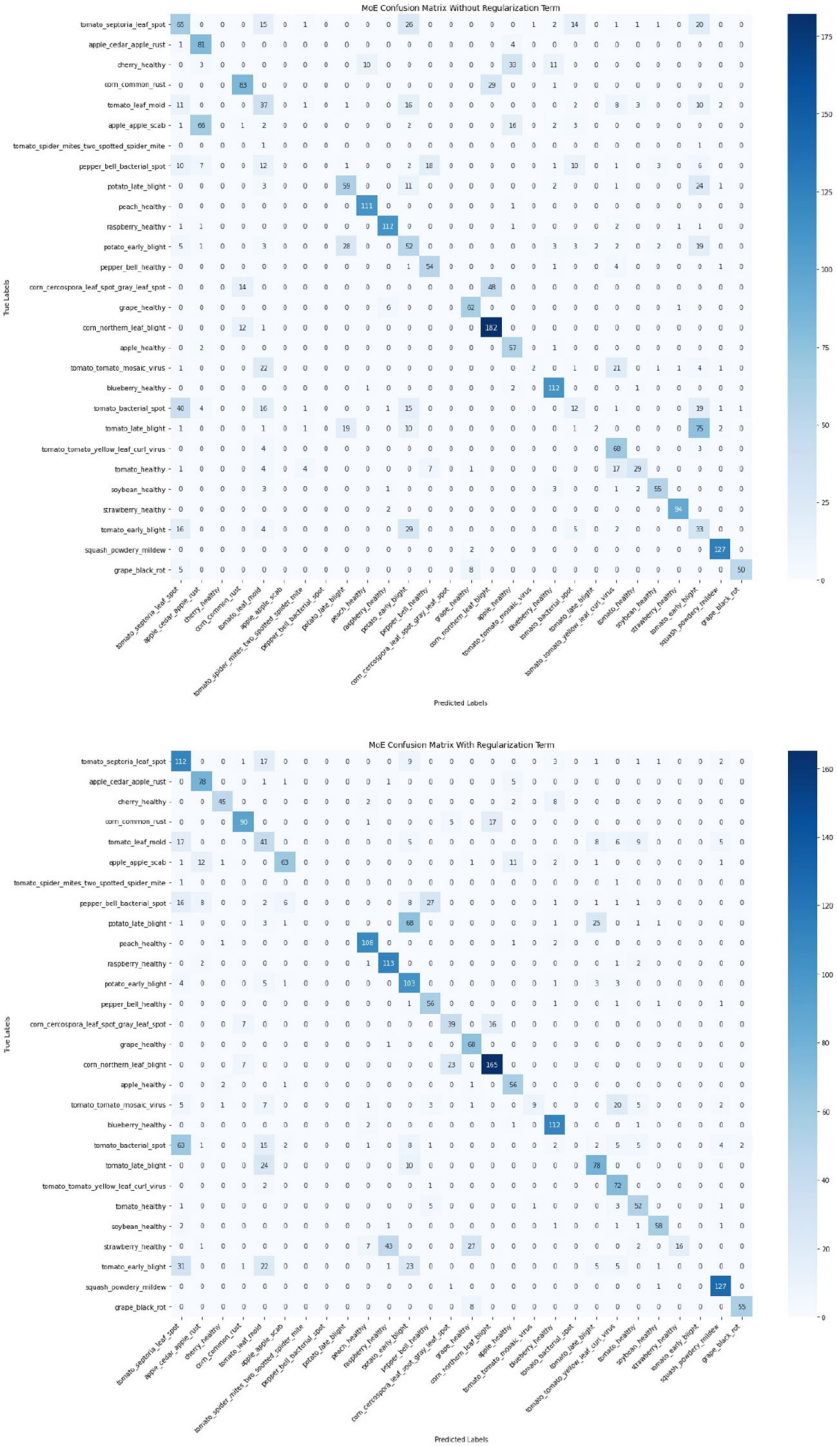
Comparison of confusion matrices before (top) and after (bottom) applying regularizations. The regularized model shows reduced misclassifications and improved class-wise accuracy, particularly for classes like tomato_septoria_leaf_spot, cherry_healthy, and tomato_early_blight, highlighting the effectiveness of the proposed regularization strategy in enhancing generalization.

**Figure 19:**
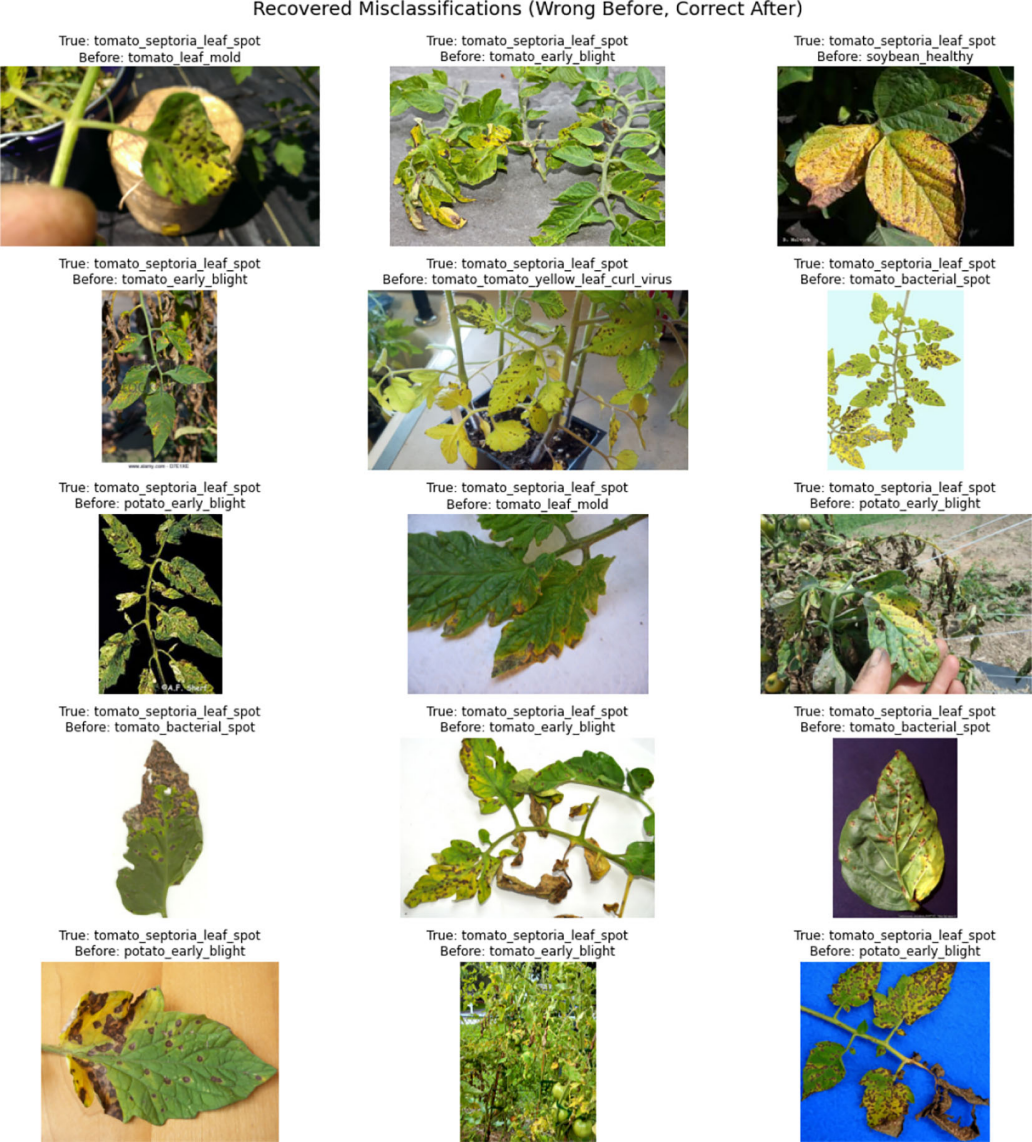
Examples of misclassified samples that were correctly predicted after applying regularization. All samples belong to the tomato_septoria_leaf_spot class. The top text indicates the true class, while the bottom shows the incorrect prediction before regularization.

To further evaluate the impact of introducing regularization terms, a comparison of confusion matrices before and after applying entropy, orthogonal, and usage regularization is presented in **Figure 18**. This comparison was conducted on the same MoE model with two configurations, i.e., without regularizations and with regularizations. The confusion matrix on the left (without regularization) displays notable misclassifications across multiple classes, whereas the confusion matrix on the right (with regularization) shows significant improvements in class-wise accuracy and reduced misclassification rates. Most prominently, the correct predictions for *tomato_septoria_leaf_spot* increased from 65 to 112, *cherry_healthy* from 0 to 45, and *tomato_early_blight* from 52 to 103. **Figure 19** illustrates the cases that were correctly classified after the introduction of regularization terms. This indicates that the inclusion of regularization terms helps the MoE framework better differentiate between visually similar classes and learn more robust class boundaries, ultimately improving overall generalization and classification stability in complex conditions.

## 6 Limitations and future work

Despite the promising results, several limitations must be addressed for broader applicability. First, the scalability of the MoE framework in handling multiple diseases within a single sample remains unexplored. In actual agricultural settings, plants often exhibit multiple diseases simultaneously, which could challenge the model’s gating mechanism, which currently outputs a single disease per input. Future research should focus on improving the model’s ability to handle multi-disease images.

The model’s applicability to new plant species is another limitation. While the approach performs well on the datasets used, its transferability to different species and diseases has not yet been tested. Agricultural contexts involve diverse plant species, each with distinct disease manifestations. To extend the model’s generalization, further exploration of transfer learning and domain adaptation techniques is needed to ensure that the model can perform well on new, unseen plant species.

Another challenge is the computational cost of the gating mechanism in the MoE framework. The MoE architecture improves accuracy and generalization by enabling specialized experts to handle diverse input conditions. However, this comes at the cost of increased computational overhead due to the evaluation of multiple experts and the gating mechanism. This additional computational load can introduce latency during inference, particularly in large datasets or real-time applications. As the number of disease types and manifestations increases, the need for more experts to handle the complexity further increases the computational demand. In resource-constrained environments, such as mobile devices or embedded systems, these factors may limit the model’s applicability. Optimizing the model for faster inference will be critical to achieving a balance between computational efficiency and performance.

Finally, while the model shows robustness to various conditions, its generalization to extreme environmental variations is uncertain. Although regularization techniques were applied to improve robustness, further data augmentation strategies or synthetic data generation could help improve the model’s ability to handle diverse and previously unseen conditions, ensuring better performance in real field applications.

## 7 Conclusions

In this research, we proposed a novel approach to plant disease classification by leveraging ViT models, enhanced with a MoE for classification, alongside entropy and orthogonal regularization. Our study focused on addressing the critical challenge related to performance degradation in plant disease detection when images that differ significantly from the controlled environments are used during training. This issue is particularly relevant in the agricultural sector, where the ability to accurately classify plant diseases in varied and unpredictable conditions is essential.

We demonstrated that our ViT-based model, when trained on a relatively small yet diverse dataset, outperformed traditional models in terms of robustness. Our experimental results indicated that the proposed model maintained high accuracy levels even when tested on datasets with different image conditions and disease severities, which are often encountered in actual scenarios.

Key findings of our research include the model’s robustness to diverse image conditions, achieved through the integration of entropy and orthogonal regularization, which enabled it to maintain high accuracy across datasets with varying image qualities, highlighting its suitability for scenarios where training data differ from testing data. Overall, this study contributes to ongoing efforts in improving plant disease detection models in the wild by providing a more adaptable and generalizable approach, which is crucial for practical deployment in the agricultural sector. Future work could focus on further optimizing the model architecture, exploring additional data augmentation techniques, and testing the model in diverse field conditions to validate its applicability in complex farming environments. The implementation details and trained models are available at https://github.com/salman32140/Vit_MoE/.

By offering a solution that effectively bridges the gap between lab-controlled datasets and complex image conditions, this research paves the way for more reliable and scalable plant disease detection systems that can support sustainable agricultural practices and enhance the role of technology in crop management strategies.

## Data Availability

Publicly available datasets were analyzed in this study. This data can be found here: https://www.kaggle.com/datasets/mohitsingh1804/plantvillage, https://www.kaggle.com/datasets/abdulhasibuddin/plant-doc-dataset.
